# Microstructural, biocompatibility and mechanical investigation of MgHAp and AgHAp: Comparative report

**DOI:** 10.1007/s10856-023-06725-3

**Published:** 2023-04-28

**Authors:** Anuradha Mahanty, Deep Shikha

**Affiliations:** Department of Chemistry, Birla Institute of Technology, Mesra, Ranchi, Jharkhand 835215 India

## Abstract

**Abstract:**

It is imperative to investigate the effect of addition of different size metallic ions in HAp and study the changes in biocompatibility and mechanical properties. Silver and magnesium ions are two vital ions needed in our body. Silver ions are known to inhibit the microbes, while magnesium ions are known to increase the mechanical properties. The present study reports the comparative properties of MgHAp and AgHAp synthesised by sol-gel wet chemical method. Changes in the morphology, phase analysis, corrosion resistance, dielectric properties, hardness and the thrombus behaviour of HAp doped Ag and Mg ions has been investigated. In this work, we have presented a comparative study of both the metal doped ionsto find which of the ions and which weight percent of the ions can be best suited to be incorporated into the HAp matrix for hard tissue implants. All wt% AgHAp showed the better corrosion resistance than all the MgHAp samples. However, MgHAp showed higher value of hardness in comparison to AgHAp samples. The mechanical strength was found to increase with the increase in Mg wt% in MgHAp but for AgHAp the hardness value decreased with increase in the concentration. The impedance and dielectric loss decreased with increasing frequency for both the samples. Both the ion doped hydroxyapatite showed moderate clotting behaviour as compared to pure HAp. But 2 wt% MgHAp and 4 wt% AgHAp showed better thrombogenic behaviour.

**Graphical abstract:**

**Figure:**
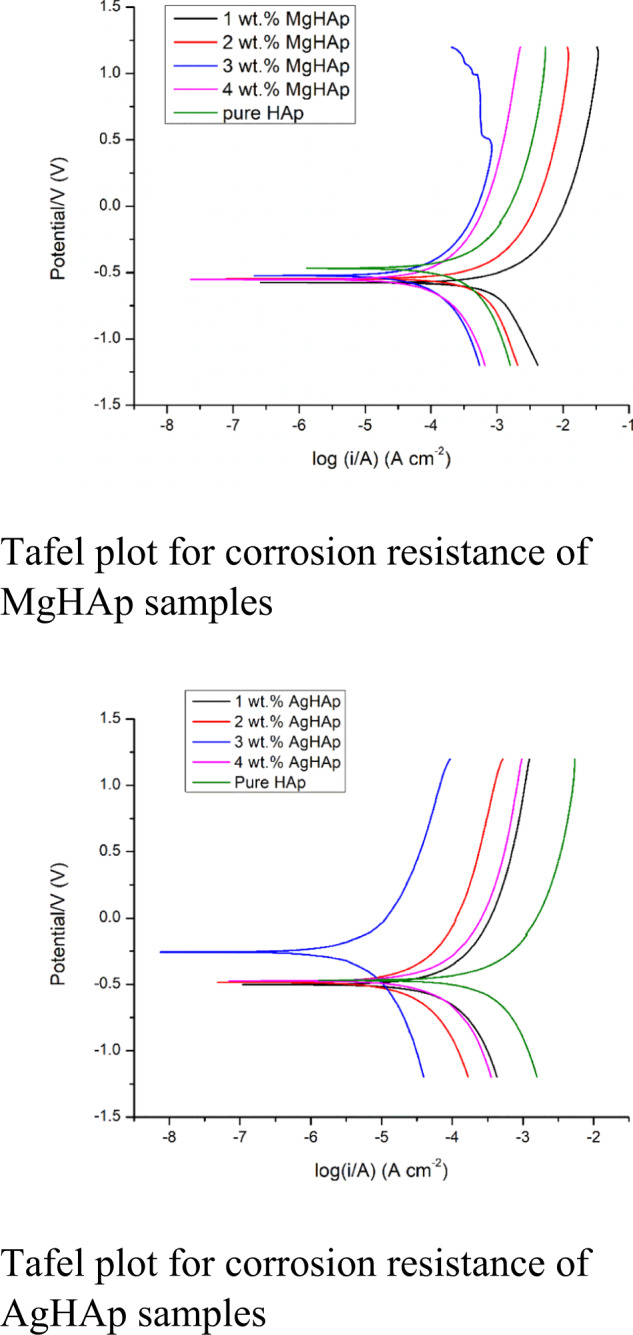
It is imperative to investigate the effect of addition of different size metallic ions in HAp and study the changes in biocompatibility and mechanical properties. Silver and magnesium ions are two vital ions needed in our body. Silver ions are known to inhibit the microbes, while magnesium ions are known to increase the mechanical properties. The present study reports the comparative properties of MgHAp and AgHAp synthesised by sol-gel wet chemical method. Changes in the morphology, phase analysis, corrosion resistance, dielectric properties, hardness and the thrombus behaviour of HAp doped Ag and Mg ions has been investigated. In this work we have presented a comparative study of both the metal doped ions to find which of the ions and which weight percent of the ions can be best suited to be incorporated into the HAp matrix for hard tissue implants. All wt% AgHAp showed the better corrosion resistance than all the MgHAp samples. However, MgHAp showed higher value of hardness in comparison to AgHAp samples. The mechanical strength was found to increase with the increase in Mg wt% in MgHAp but for AgHAp the hardness value decreased with increase in the concentration. The impedance and dielectric loss decreased with increasing frequency for both the samples. Both the ion doped hydroxyapatite showed moderate clotting behaviour as compared to pure HAp. But 2 wt% MgHAp and 4 wt% AgHAp showed better thrombogenic behaviour.

## Introduction

Due to their bioactivity and biocompatibility, calcium phosphate-based (Ca-Ps) bioceramics are employed as synthetic bone substitutes. Different phases of calcium phosphate bioceramics are known to exist. The bones and teeth of animals contain the naturally occurring calcium phosphate bioceramics hydroxyapatite (HAp) and beta tricalcium phosphate (β-TCP). Given that HAp has excellent bone-bonding properties and -TCP has strong bioresorbability in physiological environments, these two phases create an exceptional combination for bone implantation [1]. Now-a-days, research has shifted to the preparation and study of inorganic material at nanoscale for both fundamental and applied research. Nanoparticles have wide range of applications such as in catalysis, electronics, sensors, biotechnology, medicines, pharmaceuticals etc. Calcium phosphate based biomaterials havewide range of applications in medicine [2]. Surgical procedures to replace a bone continues to hold place in the field of orthopaedics dealing with skeletal defects. Autologous bone grafting is still treated as a standard treatment for bone injuries. In order to avoid donor sites and realising off-shelf convenience has led to the use of biomaterial bone substitutes. The first-generation materials were used for cranial reconstruction and was specifically designed for biomedical implants as described in the paper by Friedman et al. [3].

Amongst the ceramics, hydroxyapatite (HAp) has been proved to be a promising candidate to be used as biomedical implant. HAp is an excellent material to be used as a hard tissue replacement because of its chemical properties and structure matching to that of human bones and teeth. Synthetic HAp is now widely finding its use in clinical applications because of its bioactivity thereby promoting bone growth directly on its surface [4]. HAp is introduced as a biomaterial in bone repair, bone implants and bone drug delivery systems in early 1990s owing to its osteoconductivity, osseointegration, bioactivity, biocompatibility and non-toxicity [5]. Ceramic biomaterials that are based on nano sized HAp are known to exhibit higher bioactivity and improved resorbability than micro sized HAp. The release of calcium ions from nano sized HAp follows the same pattern as that of the natural apatite and is faster than the coarse grained crystals [6]. Biocompatibility studies of HAp has been characterized by minimal inflammatory response with no foreign body response and no giant cell reactions. The reason being, the only metabolites in HAp are calcium and phosphates ions [7].

Pure HAp crystallises in a hexagonal lattice. Three different ions, Ca^2+^, PO_4_^3−^, and OH^−^ are present in stoichiometric HAp, which is one of the key structural characteristics. HAp contains both Ca(I) and Ca(II) ions. Ca(I) ions are found along the hexagonal unit cell borders, whereas Ca(II) ions arrange themselves into equilateral triangles with hydroxyl groups in the centre. The oxygen atoms are positioned so that they cannot establish a hydrogen bond with the OH- groups at the borders of the elementary cells, generating columns of OH-OH-OH-. The biggest ions, the PO_4_^3−^ ions, are the ones that dictate the structure of HAp [8]. The axis of O and Ca atoms are parallel to the hexagonal axis with the cage constants a = 0.9418 nm and c = 0.6884 nm forming hexagonal structure of stoichiometric HAp [Ca_10_(PO_4_)_6_(OH)_2_]. Furthermore, calcium-deficient HAp [CDHAp, Ca_10-x_(PO_4_)_6-x_(HPO_4_)_x_(OH)_2-x_; 0 ≤ x ≤ 1] ismore of biological concern than stoichiometric HAp as the Ca/P ratio of the bone is close to 1.5 [6].

The ability of HAp to serve as an implant is subjected to some restrictions. These include undesirable mechanical characteristics like rigidity, brittleness, and low fracture toughness [9, 10]. HAp is known to be very brittle ceramic and possessing very strong compression and weak tensile strength [11]. In addition to the aforesaid, the rate of osseointegration in HAp is slow [10]. Recently, HAp is used as implant material due to its excellent osteoconductive property, which supports osseointegration and osteogenesis processes [12]. The development of fungi and bacteria on the surface of HAp over time renders it unfit for use as a long-term implant. We must thus move to materials that can overcome these drawbacks. Since HAp is prone to ion-exchange processes, as was already noted, other dopants, such as metal ions, non-metal ions, and polymers, can be added to HAp to enhance its performance [8]. Metal ions like silver, copper, zinc, magnesium etc. are known to exhibit antimicrobial properties. These dopants widely find their usage in in-vitro and in-vivo applications. Silver is known to prevent bacterial colonisation of prostheses, catheters and human skin [13].

Due to their minimal toxicity to human cells, silver ions are of considerable interest. When integrated into the HAp matrix, biomaterials containing silver have Ag^+^ ions in their elemental state. A new biomaterial with improved osteoconductivity, good biocompatibility, new bone-forming capacity and antimicrobial characteristics is created when HAp is substituted with other ions. Magnesium, on the other hand, is a vital mineral for many body processes and is one of the seven important microminerals. It is necessary for the development of strong bones. Mg^2+^ ions are known to promote osteoblast proliferation in the initial phases of osteogenesis. Magnesium deficiency has effects on skeletal metabolism, including improper bone formation, decreased osteoblast activity and weak bone. Lack of magnesium may cause osteoporosis [8]. In the HAp lattice, magnesium is a well-known cationic substituent. Magnesium ions support osteoblast and osteoclast functions, which aid in the mineralisation of bone. The amount of Mg added inhibits the development and stabilisation of more acidic precursors while also inhibiting HAp nucleation. High magnesium concentrations in HAp also resulted in decreased crystallinity, increased incorporation of HPO_4_^2−^ and increased dissolution extent. With higher amount of magnesium present, the size of the lattice clearly reduced [14]. The incorporation of Mg^2+^ ions within the HAp structure is essential for developing artificial bone substitutes [15].

The present study aims on synthesis and characterization of doping different weight percent of magnesium and silver in the HAp matrix for artificial bone implants. Different weight percent of Mg and Ag ions were taken and a comparative study amongst them were carried out to check which of the ions and what weight percent can be best suited to act as a hard tissue implant. The weight percent taken for the present study was 1, 2, 3 and 4 wt% which has not been previously studied. The Sol-gel wet chemical method was used for the synthesis of HAp, MgHAp and AgHAp samples. The Sol-gel technique is preferred due to its cost effectiveness, low synthesis temperature, homogenous mixing, high purity of the products and formation of nano-sized particles. The Ca/P, (Ca+Mg)/P and (Ca+Ag)/P ratio for pure HAp, MgHAp and AgHAp samples respectively was maintained in the range of 1.5 to 1.68. Furthermore, the characterization was done using X-Ray diffraction (XRD) to analyse the phase, the crystallite size and the lattice cell parameters. The formation of HAp was confirmed with the hkl values and was matched with the JCPDS software. The various functional group detection and their shifting was studied through Fourier Transform Infrared Spectroscopy (FTIR). The surface morphology and shape of the grains was studied using Field Emission Scanning Electron Microscopy (FESEM) coupled with EDX. The amount of Mg and Ag incorporated quantitatively was checked using Inductively Coupled Plasma-Optical Emission Spectrometry (ICP-OES). The various modes of phosphate vibration and the site of incorporation of Mg and Ag into HAp matrix was studied by Raman Spectroscopy. The human body contains various fluids. The implant needs to sustain the body temperature of 37 °C. The ions present in the body fluids can lead to corrosion of the implant.Therefore, it is very important to check the corrosion resistance of the samples. The corrosion activity of both types of the samples were checked in Ringer’s solution.The dielectric properties of the samples were checked from the impedance studies using Bode’s plot. The average pore radius and surface area was checked from Brunauer–Emmett–Teller (BET) studies. Thrombogenic studies using whole blood clotting assay were carried out to check the clotting behaviour of the samples. Finally, a comparative study was done in order to evaluate the best weight percent Ag or Mg doped sample for its application as future orthopaedic implant.

## Materials and methods

### Materials

All the chemicals used in the experiment were of analytical grade. Ca(NO_3_)_2_.4H_2_O (Fischer Scientific)_,_ (NH_4_)_2_HPO_4_ (Merch), Mg(NO_3_)_2_.6H_2_O (Fischer Scientific), AgNO_3_ (Qualigens) absolute ethanol (≥99.5% ACS, ISO reagent, Merch), Millipore water and NH_3_ solution (99.98%, Merch) were used as the starting materials for the synthesis of pure HAp, MgHAp and AgHAp samples.

### Synthesis of Pure HAp

Pure HAp was synthesised using Ca(NO_3_)_2_.4H_2_O and (NH_4_)_2_HPO_4_ as the Ca and P precursors, respectively. The solvent used was absolute ethanol. The Ca precursor solution was prepared by constant stirring the Ca precursor in absolute ethanol for 2 h. The P precursor solution was prepared by constant stirring the P precursor in Millipore water followed by the addition of absolute ethanol for 2 h. The Ca/P ratio was maintained at1.67 by slow addition of P solution in the burette to the Ca solution by constant stirring for 6 h at normal temperature. The sol was converted to gel by increasing the pH of the solution to 10 by addition of ammonia to the sol. The solution was given for ageing for 24 h which was followed by oven drying at 100 °C until complete dry. The dried sample was crushed using a mortar pestle until fine powder followed by sintering at 680 °C for 3 h in muffle furnace. The pellets of the sintered powder were prepared using a KBr die set in hydraulic press at a pressure of 20 kg/cm^2^. 13 mm diameter pellets were prepared. This was followed by sintering of the pellets at 680 °C for 3 h.

### Synthesis of Mg fabricated HAp (MgHAp)

For the synthesis of MgHAp; Ca(NO_3_)_2_.4H_2_O, (NH_4_)_2_HPO4 and Mg(NO_3_)_2_.6H_2_O were taken as the Ca, P and Mg precursors, respectively. Absolute ethanol was taken as solvent. The Ca precursor solution was prepared by constant stirring the Ca precursor in absolute ethanol for 2 h. The P precursor solution was prepared by constant stirring the P precursor in Millipore water followed by addition of absolute ethanol for 2 h. The Mg precursor solution was prepared by constant stirring of Mg precursor in absolute ethanol for 2 h. This was followed by mixing the Mg precursor solution to the Ca precursor with constant stirring for 2 h to form (Ca+Mg) solution. The (Ca+Mg)/P ratio was maintained to 1.67 by slow addition of P precursor solution in the burette to the (Ca+Mg) solution by constant stirring for 6 h at normal temperature which resulted in the formation of sol. The sol was converted to gel by increasing the pH of the solution to 10 by addition of ammonia to the final solution. The solution was aged for 24 h followed by drying the solution at 100 °C in the oven until complete dry. The dried sample was crushed until fine powder using a mortar pestle. The crushed fine powders of MgHAp were given for sintering at 680 °C for 3 h in muffle furnace. The pellets of the sintered powder were prepared using a KBr die set in a hydraulic press at a pressure of 20 kg/cm^2^. The diameter of the pellets prepared was 13 mm. The MgHAp pellets was given for sintering at 680 °C for 3 h in muffle furnace. This was followed by the characterization of the MgHAp pellets.

### Synthesis of Ag doped HAp

For the synthesis of AgHAp, Ca(NO_3_)_2_.4H_2_O, (NH_4_)_2_HPO_4_ and AgNO_3_ were taken as the Ca, P and Ag precursors respectively. Absolute ethanol was taken as solvent. The Ca precursor solution was prepared by constant stirring the Ca precursor in absolute ethanol for 2 h. The P precursor solution was prepared by constant stirring the P precursor in Millipore water followed by addition of absolute ethanol for 2 h. The Ag precursor solution was prepared by constant stirring of Ag precursor in absolute ethanol for 2 h. This was followed by mixing the Ag precursor solution to the Ca precursor with constant stirring for 2 h to form (Ca+Ag) solution. The (Ca+Ag)/P ratio was maintained to 1.67 by slow addition of P precursor solution in the burette to the (Ca+Ag) solution by constant stirring for 6 h at normal temperature which forms the sol. The sol was converted to gel by increasing the pH of the solution to 10 by addition of ammonia to the final solution. The solution was aged for 24 h which followed by drying the solution at 100 °C in the oven until complete dry. The dried sample was crushed until fine powder using a mortar pestle. The crushed fine powders of AgHAp were given for sintering at 680 °C for 3 h in muffle furnace. The pellets of the sintered powder were prepared using a KBr die set in a hydraulic press. The diameter of the pellets prepared was 13 mm at a pressure of 20 kg/cm^2^. The AgHAp pellets were sintered at 680 °C for 3 h in muffle furnace. This was followed by the characterization of the AgHAp pellets.

### Characterizations

The compound formation and the phase analysis were carried out using XRD studies by powder X-Ray diffractometer (Rigaku, Japan, Smart Lab 9 kW) using CuK_α_ radiation (0.154 nm) with step size of 0.01 in the 2θ range from 10° to 70° in a continuous scan mode. The vibrational modes of pure HAp, MgHAp and AgHAp pellets was checked using FTIR (Shimadzu Corpn., Japan, IR-Prestige 21) in the Attenuated Total Reflectance (ATR) mode ranging from 4000–400 cm^−1^. The morphology and elemental analysis of HAp, MgHAp and AgHAp was done using FESEM coupled with EDX system (Sigma 300, Zeiss, Germany). The average pore radius and the surface area of the pore was evaluated using BET (Autosorb, Quanta chrome, USA). The potentiodynamic polarisation was investigated by dipping the samples in Ringer’s solution. The Ringer’s solution was prepared using the composition; NaCl (9 gm), KCl (0.42 gm), CaCl_2_ (0.48 gm) and NaHCO_3_ (0.2 gm) in 1 L distilled water at 37 °C. The Tafel plot was obtained using Electrochemical Analyser (CH Instruments, USA, Model 680B) with Potential/V on the *Y*-axis and log (Current/A) on the *X*-axis.

#### Mechanical studies

The mechanical properties of pure HAp and various weight percent MgHAp & AgHAp were evaluated using the hardness test. The hardness test was performed using Vicker’s micro-indenter. All the samples were tested for their hardness. During the test, a force of 200 gf and 300 gf with a dwelling time of 15 s was applied to ensure that no cracks were seen. Each sample were tested for their hardness at three different locations. The average value of the hardness was computed and reported.

#### Whole blood clotting method

The thrombogenicity of the samples was carried out using the whole blood clotting assayas mentioned [16]. 2 mL of fresh blood from healthy human was extracted. It was immediately transferred to the ethylene diamine tetraacetate (EDTA) box to prevent clotting. 5 replicates of the same sample were placed in different petri dish. The EDTA-blood complex was taken in a beaker and activated using 0.0055 g (0.1 M) CaCl_2_ at the start of the experiment. 30 µL of the activated blood was added to the sample. The samples were incubated for 5, 15, 25, 35 and 45 min, respectively, in an incubator at 37 °C. 5 mL of distilled water was added to each of the sample after incubation. After addition of distilled water, each of the samples were incubated for 10 min. The red blood cells that were not trapped in a thrombus were lysed with the addition of distilled water, thereby releasing haemoglobin (Hb) into the water for subsequent measurement. The blood which was not lysed was filtered out in a conical flask. Absorbance of the solution was measured at 540 nm using a Spectrophotometer (Labindia Analytical UV 3200, UV/VIS Spectrophotometer). Absorbance value was found to be less for more clotting.

## Results and discussions

### X-Ray Diffraction (XRD) analysis

**Fig. 1:**
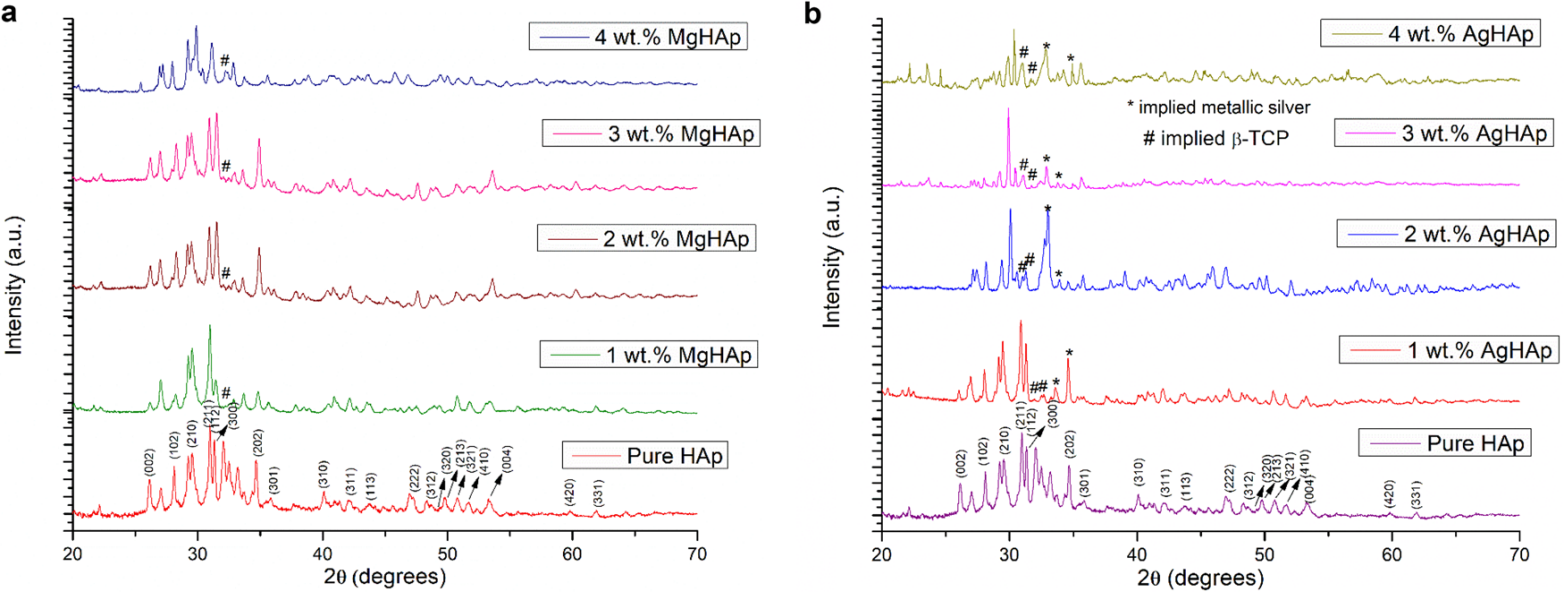
**a** Represents the XRD graph of MgHAp samples. **b** Represents the XRD pattern for AgHAp samples

The XRD pattern for pure hydroxyapatite and different doped wt% of MgHAp & AgHAp are shown in the Fig. 1a,b, respectively. The miller indices were obtained using Rietveld refinement of the XRD data taken over range of 2θ~ 10° to 70° using Full prof suite software. The XRD study revealed that the phase for pure hydroxyapatite was hexagonal with the space group P6_3_/m, JCPDS file no.09-432 which was the main apatite phase and the sharp peaks showed that they are well crystallised [17]. The miller indices corresponding to pure HAp are (002), (102), (210), (211), (112), (300), (202), (301), (310), (311), (113), (222), (312), (320), (213), (321), (410), (004), (420) and (331) [18]. The XRD pattern of HAp and different weight percent of MgHApis presented in Fig. 1a.

For 1 wt% MgHAp, there was decrease in the intensity of the peak present at 2ϴ = 32.1° (112) [19]. Broadening of diffraction peaksis caused due to the lattice strains as Mg atoms are incorporated in the HAp lattice [20–22]. The peak shift is caused by the partial formation of MgHAp which has merged reflection in the 2ϴ range at 29° to 33° and 45° to 54° [14]. The relative intensity of the peak corresponding to 2ϴ = 54° has decreased with increase in the wt% of Mg. As the wt% of Mg increases, XRD diffraction shows shifting towards higher angle with respect to pure HAp [23]. The incorporation of 2 wt% MgHAp resulted in partial substitution of Mg^2+^ ions by the Ca^2+^ ions which is evident from shifting of (002) X-Ray reflection [24]. On comparing the different wt% MgHAp, the low intensity of the adjacent peaks could be attributed to low degree of crystallisation in the range of 35°–38° for 1 and 4 wt% MgHAp but for 2 wt% and 3 wt% show high intensity peaks are attributed to the high degree of crystallisation when compared to pure HAp [25]. With the increase in wt% of Mg, relative intensity of the peaks decreases and overlapping of the main peaks occurs [26]. It could possibly be explained due the decreased crystallite size and increased disorder in the lattice as wt% of Mg increases in the HApmatrix [27–29]. Therefore, crystallite size is calculated using the Scherrer’s formula:

$$D = K\lambda /\beta _{1/2}cos\theta$$

Where; K is the Scherrer’s constant, λ is the wavelength of incident X-Ray beam, β_1/2_ is the full width half maxima for the diffraction peak and ϴ is the diffraction angle. It is clear from Table 1a that as the wt% of the dopant increases, the crystallite size decreases.

**Table 1:** a: The crystallite size of MgHAp samples as calculated from the Scherrer’s formula. b: The crystallite size of AgHAp samples as calculated from the Scherrer’s formula. c: The lattice parameters and cell volume computed for different weight percent of MgHAp. d: The lattice parameters and cell volume for different weight percent of AgHAp samples

Sample Name	Crystallite Size (nm)
a	
Pure HAp	39.36
1 wt% MgHAp	26.81
2 wt% MgHAp	24.18
3 wt% MgHAp	20.62
4 wt% MgHAp	17.89
b	
Pure HAp	39.36
1 wt% AgHAp	42.07
2 wt% AgHAp	44.66
3 wt% AgHAp	47.28
4 wt% AgHAp	52.90

Name of the sample	a (Ǻ)	c (Ǻ)	Cell volume (Ǻ^3^)
c			
Pure HAp	9.425	6.882	529.41
1 wt% MgHAp	9.389	6.887	525.758
2 wt% MgHAp	9.351	6.883	521.208
3 wt% MgHAp	9.347	6.798	514.331
4 wt% MgHAp	9.338	6.754	510.019
d			
Pure HAp	9.425	6.882	529.41
1 wt% AgHAp	9.433	6.886	530.62
2 wt% AgHAp	9.446	6.891	532.47
3 wt% AgHAp	9.450	6.899	533.54
4 wt% AgHAp	9.452	6.903	534.07

The XRD pattern of pure HAp and different weight percent of AgHAp is presented in Fig. 1b. Substitution of Ag in HAp lattice shows the presence of sharp peak in the range of 33° to 34° for 1, 2 and 4 wt%. In case of 3 wt% AgHAp, the intensity of metallic silver peaks as represented in asterisk (*) was less [30]. Due to the larger radii of Ag^+^ ions, there is residual stress and defect concentration resulting in shifting of peaks for all the AgHAp samples [31]. There is a subsequent increase of crystallite size with the increase in the wt% of Ag in HAp as calculated from the Scherrer’s formula. Contrary to MgHAp, the degree of crystallinity, lattice parameter and crystallite size in AgHAp increases with increase in the wt% of Ag, implying that the substitution of Ag^+^ on Ca^2+^ [Ag^+^ (0.128 nm)> Ca^2+^ (0.099 nm)] has resulted in the increase in the lattice parameter of all the AgHAp samples [32].

The β-TCP phase starts to form around 600 °C and HAp structure completely transforms into the calcium deficient HAp (β-TCP) at around 1000 °C [33]. The β-tricalcium phosphate (β-TCP) phase was identified between 2ϴ = 30.05° to 32.66° as marked with # sign in Fig. 1a,b. The increase in calcination and sintering temperature will increase the ratio of TCP phase [34]. The Ca/P molar ratio was found to be less than 1.67. This inferred that the β-TCP phase has increased during thermal treatment [35]. All peaks are of HAp phase except two minor peaks around 2ϴ = 30.05° to 32.66° are of β-TCP phase. Also, with the increasing molar ratio, β-TCP phases start to disappear [36]. Addition of Mg^2+^and Ag^+^ resulted in the biphasic structure comprising HAp as the major phase and β-TCP as the secondary phase which increases with the dopant ion concentration.

The crystallite size as calculated from the Scherrer’s formula is presented in Table 1b. The data presented in Table 1b shows that as the Ag concentration is increased, the crystallite size also increased which is contrary to MgHAp.

The lattice parameters and the cell volume for MgHAp samples are listed in the Table 1c. Reduction in lattice parameter is due to the substitution of smaller Mg^2+^ ion (0.72 Ǻ) in place of Ca^2+^ (0.99 Ǻ) [37]. Addition of Mg considerably decreased the overall crystallinity of HAp. Mg^2+^ ion substitution is essentially happening in the Ca(I) site and not in the Ca(II) sites. This is because in the Ca(I) sites, the oxygen atoms are arranged in distorted trigonal prism [25]. Increasing the Mg^2+^ ion concentration which implied ionic substitution of Ca^2+^ by smaller ion Mg^2+^ resulted in the contraction of cell parameters of HAp as shown in Table 1(c). Decrease in the stability of HAp doped with Mg was found due to more ionic substitution of Mg ions in the Ca^2+^ matrix [27]. The change of lattice parameter in the ‘a’ plane is slightly larger than the ‘c’ plane; which in turn strengthened the substitution of Mg^2+^ ions in the Ca^2+^ ions in the a/b plane along with the formation of shorter Mg-O bonds [38].

The lattice parameters and cell volume for AgHAp are listed in Table 1d. Change in lattice parameters imply that Ag^+^ ion has been substituted Ca^2+^ ion in the HAp lattice.Since Ag^+^ (0.128 nm) has radii larger than Ca^2+^ (0.099 nm), there is a subsequent increase in the lattice parameters ‘a’ and ‘c’ [39]. Ag gets fully substituted in the HAp matrix with increase in the Ag concentration [40]. Ag^+^ ion could be partially substituted in the Ca^2+^ sites andhas the possibility to change the crystalline environment. Ag^+^ ion might occupy the lattice or the interstitial sites depending on the amount of silver incorporated [41]. There is subsequent increase in the cell volume due to the increase of lattice parameter of ‘a’ and ‘c’ values.

### Fourier Transform Infrared Spectroscopy (FTIR) analysis

**Fig. 2:**
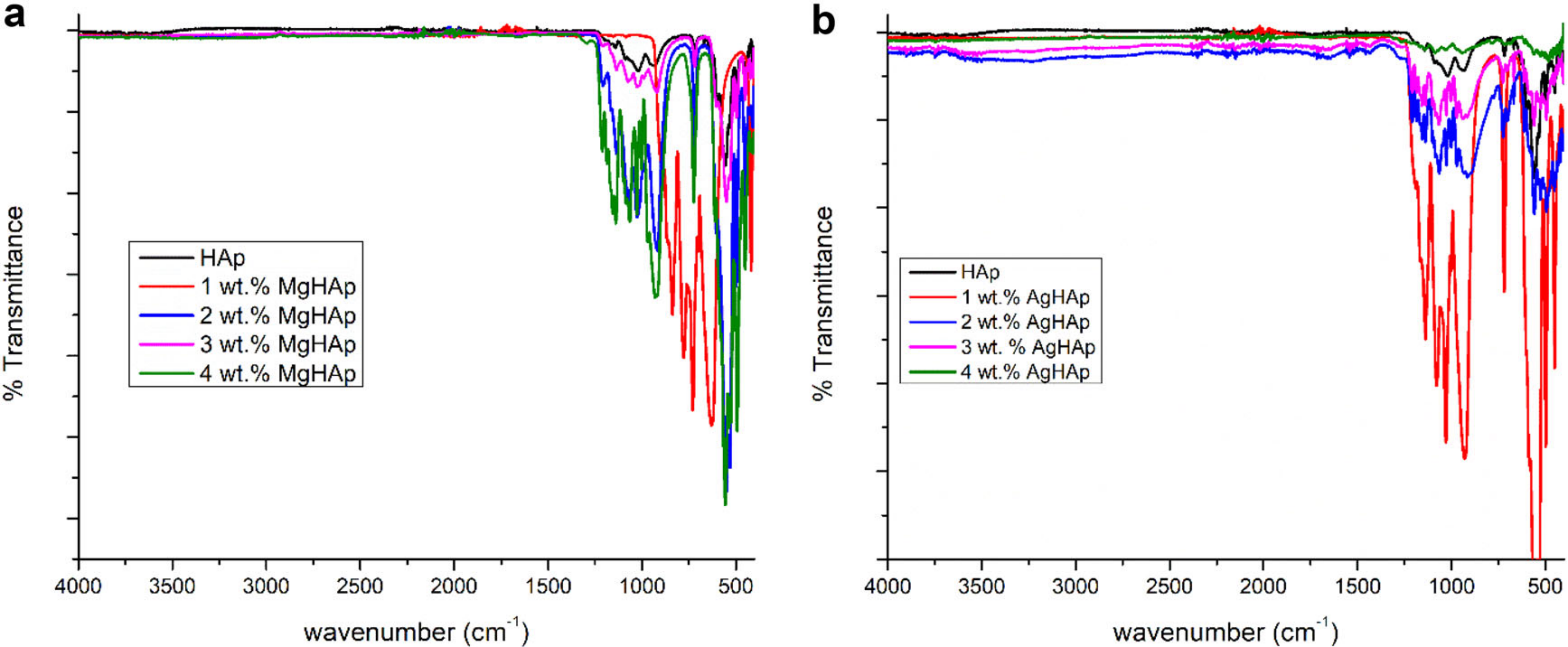
**a** The FTIR spectra of pure HAp and all weight percent MgHAp. **b** The FTIR spectra of pure HAp and all weight percent AgHAp samples

The FTIR spectra as shown in Fig. 2a for MgHAp shows OH^-^ stretching vibration at 3565 cm^−1^. The phosphate stretching vibration is seen at 1032, 927 and 923 cm^−1^. The bending modes of phosphate groups are seen at 472, 565 and 490 cm^−1^. The carbonate groups are seen at 1461, 1423 and 875 cm^−1^. It is observed that the intensity of the peaks decreased as Mg concentration is increased. This is because Mg inhibits the crystal growth which is confirmed by the XRD data.

The spectra as shown in Fig. 2b reveal theFTIR of HAp and all the weight percent of Ag doped hydroxyapatite. The various functional groups detected were OH^−^, PO_4_^3−^, CO_3_2^−^ and adsorbed H_2_O. The OH^-^ stretching vibration for silver doped HAp was seen at 3740 cm^−1^. The bending vibration of OH^-^ was found at 1600–1700 cm^−1^ [42]. The CO_3_2^−^ groups are seen at 1427 cm^−1^. The PO_4_^3−^ groups stretching vibration are seen around 1060, 932, 941.7, 943.39 cm^−1^ while the bending vibration of PO_4_^3−^ groups are seen at 495.99, 605, 559 and 665 cm^−1^ [39]. The difference seen between MgHAp and AgHAp is the shifting of OH group from 3565 to 3740 cm^−1^. Another difference between MgHAp and AgHAp FTIR plot is that in finger print region the absorption decreases with the increase in wt% of Ag in AgHAp. This may be due to the increased crystallite size and volume of AgHAp samples.

### Inductively coupled Plasma Optical Emission Spectrometry (ICP-OES)

The total amount of magnesium and silver incorporated in the HAp lattice was quantitatively checked by the Inductively coupled Plasma Optical Emission Spectrometry. The amount of magnesium and silver ions present in each sample was listed in Table 2a, b. It is observed that with the increase in wt%, ICP also shows the increase in concentration which confirms the incorporation of the dopant in the HAp matrix.

**Table 2:** a The weight percent of Mg present in the MgHAp. b The weight percent of Ag present in the AgHAp

S.No.	Name of the sample	Weight percent of Mg present
a		
1.	1 wt% MgHAp	0.0112
2.	2 wt% MgHAp	0.0210
3.	3 wt% MgHAp	0.0320
4.	4 wt% MgHAp	0.1080

S.No.	Name of the sample	Weight percent of Ag present
b		
1.	1 wt% AgHAp	0.0410
2.	2 wt% AgHAp	0.0485
3.	3 wt% AgHAp	0.0537
4.	4 wt% AgHAp	0.1139

### Raman spectroscopy

**Fig. 3:**
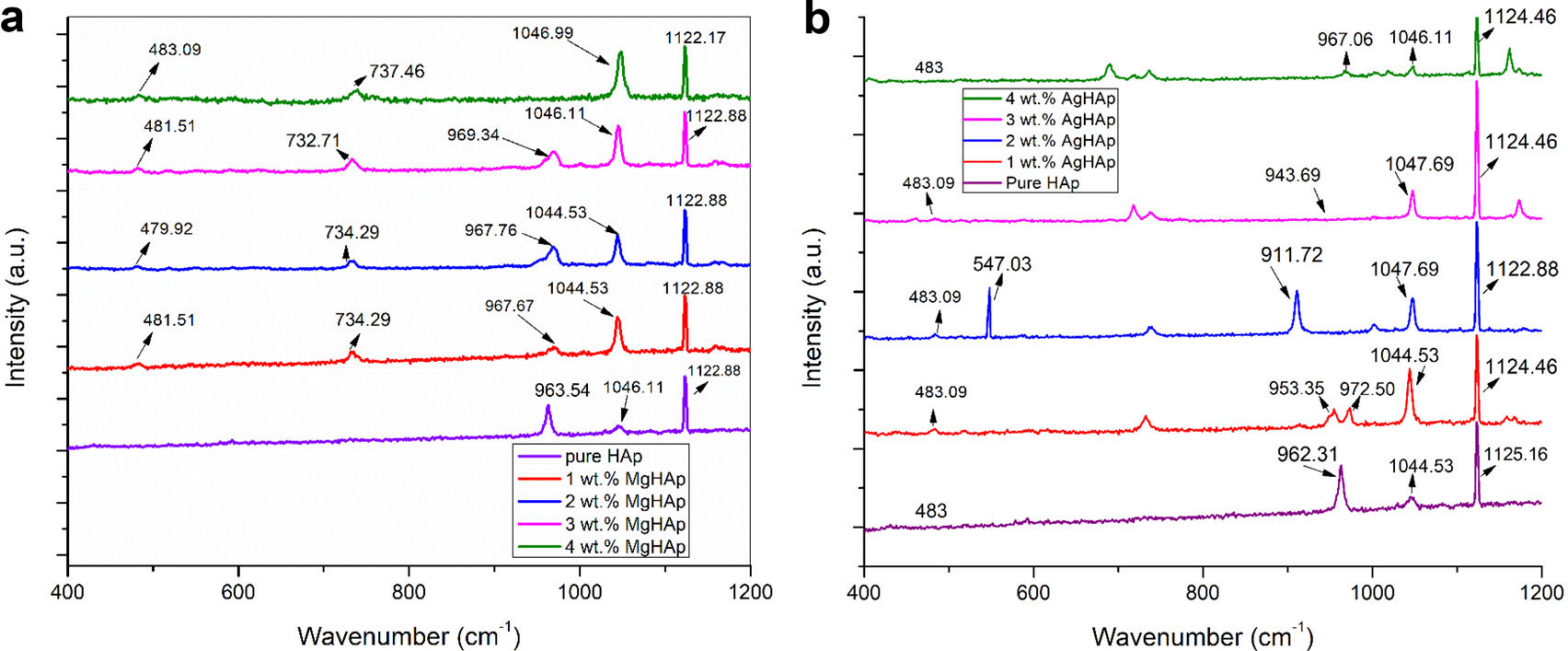
**a** The Raman spectra of MgHAp samples. **b** The Raman spectra of AgHAp samples

The Raman spectra for different weight percent of MgHAp is presented in Fig. 3a. Diallo-Garcia et al. [43] used Raman spectroscopy to investigate the efficacy of adding Mg^2+^ to the structure as well as the presence of other phases which is consistence with our explanation. The main peak for the ν_1_ vibration mode of PO_4_^3−^ ranged from 963.54 to 969.34 cm^−1^ in all samples with varying concentration of Mg^2+^. The emergence of a new vibration band with a frequency around 734.29 to 737.46 cm^−1^ may be the cause of the broadening of XRD. Mg may have been substituted for Ca^2+^ in the HAp lattice, or Mg-HAp may have broken down producing magnesiumsubstituted -TCP (Ca_(3-z)_Mg_z_(HPO_4_)_x_(PO_4_)^2-^_(x-3)_) [44]. Additionally, the band seen around 1044.53–1046.99 cm^−1^ for all MgHAp concentrations confirmed the PO_4_^3-^ peaks ν_3_ stretching mode. For pure HAp and all wt% of MgHAp, the symmetric stretching mode of the PO_4_^3-^ peak was observed around 1122.17 to 1122.88 cm^−1^, respectively.This shifting of peak towards lower energy reveals the incorporation and stability of MgHAp samples. The faint peaks at 479.92–483.09 cm^−1^ demonstrate the PO_4_^3-^ν_2_ bending modes. The amount of calcium that can be replaced by magnesium is constrained by the substantial size difference between Mg^2+^ and Ca^2+^. This variation causes a significant distortion in the crystal lattice of HApwhich eventually lowers the crystallinity [45].

The Raman spectra of different weight percent AgHAp samples are shown in Fig. 3b. A very dense ν_1_ (PO_4_^3−^) band corresponding to the symmetrical P-O strain mode for free phosphate ions is observed at 911.72 cm^−1^ for 2wt% AgHAp [46]. Other phosphate bands found were asymmetrical P-O vibration band appearing 1044.53–1047.69 cm^−1^ for ν_3_ (PO_4_^3−^) and O-P-O bending vibration (ν_4_ PO_4_^3−^) occurring at 547.03 cm^−1^ for 2 wt% AgHAp. Raman spectra at 547.03 cm^−1^ is seen when Ag (x) concentration is between 0.0 ≤ x ≤ 2.0 [Ca_10-x_ Ag_x_(PO_4_)_6_(OH)_2_] [47]. 1 wt% AgHAp composition showed the presence of two peaks at frequencies of 953.35 and 972.50 cm^−1^, which correspond to HAp (major) phase and TCP phase (minor), respectively [48]. The low intensity peak around 483 cm^−1^ corresponds to the doubly degenerate bending phosphate mode for 1–3wt% and diminishes in 4 wt% AgHAp [49]. However, peaks at 911.72 and 547.03 cm^−1^ are not observed for MgHAp samples which may imply that MgHAp samples are most stable than AgHAp samples.

### Mechanical studies

**Fig. 4:**
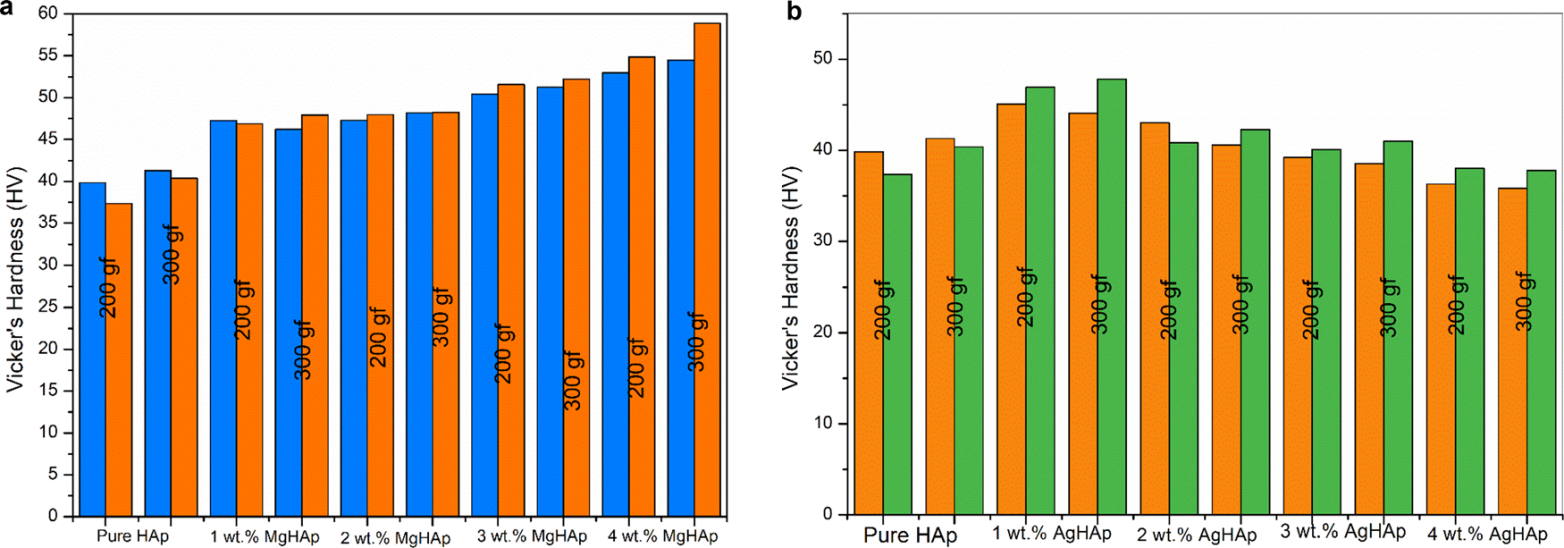
**a** The Vicker’s Hardness test for MgHAp samples. **b** The Vicker’s Hardness for AgHAp samples

Figure 4a, b show the hardness bar chart of MgHAp and AgHAp samples respectively. Increase in the hardness is beneficial for cell attachment and proliferation [50]. Dopants enhance surface area and decreases the crystallite size, which drives the densification and resulted in greater mechanical characteristics. Dopants of smaller size when substitutes the Ca sites, the bond length decreases and bond strength increases. As a result, the samples became tougher and more compact. Higher loads resulted in less plastic flow in the material, which led to increase in the resistance of the material [51]. In case of MgHAp samples, as the Mg^2+^ concentration increases, the crystallite size decreases therefore its hardness increases whereas in case of AgHAp samples, with the increase in the Ag^+^ concentration, the crystallite size increases and hence hardness decreases [51] which is in agreement with our XRD results.

### Electrochemical corrosion analysis

**Fig. 5:**
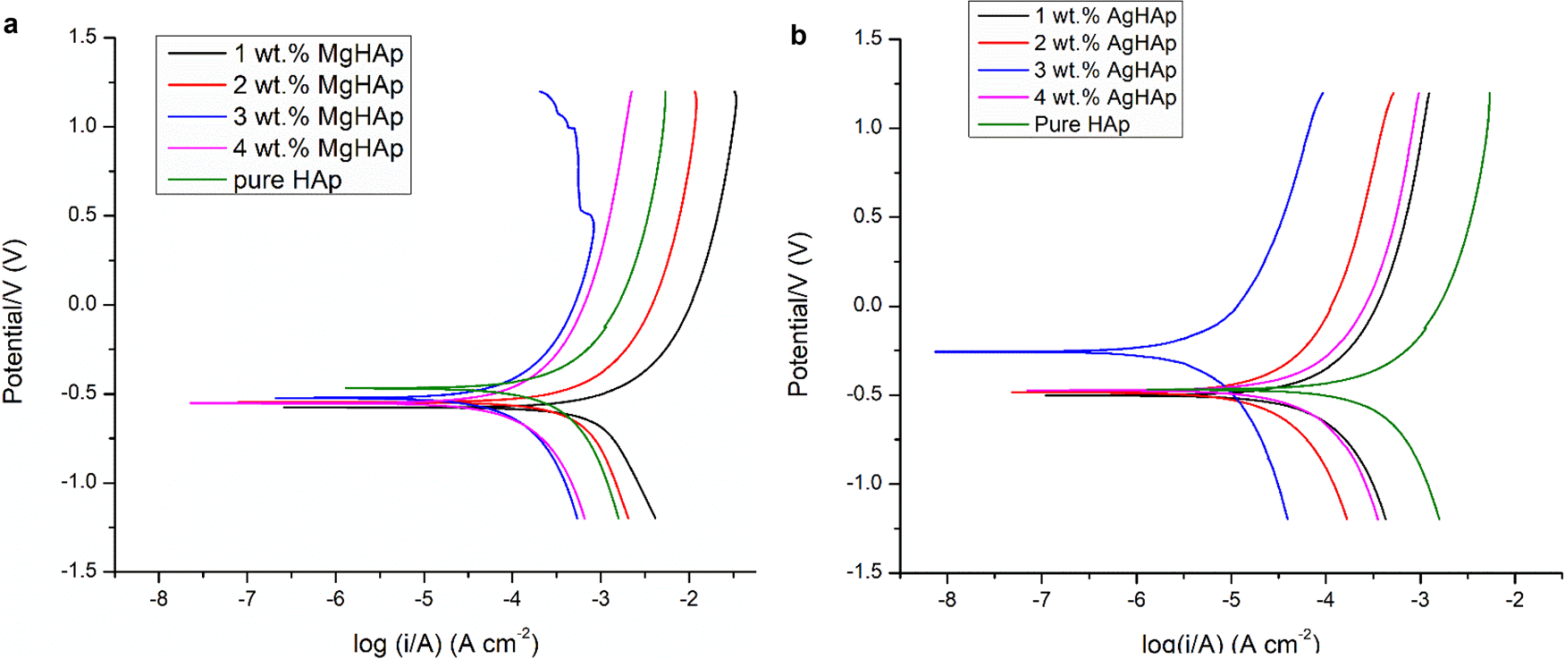
**a** The combined Tafel plot for MgHAp samples. **b** The combined Tafel plots for AgHAp samples

The main problem associated with the failure of the most of the implant is its inability to resist corrosion. Therefore, it is very important to check the corrosion behaviour of the samples. The corrosion analysis helps in assessing the biocompatibility of the samples. The tendency of the samples to corrode was checked by dipping the samples in the Ringer’s solution. The Tafel plot for MgHAp is presented in Fig. 5a. From the extrapolation of the Tafel plot, the corrosion potential (E_corr_) and corrosion current (I_corr_) are obtained. The measured E_corr_ & I_corr_ values are listed in Tables 3a, b for MgHAp and AgHAp samples respectively. An electropositive corrosion potential, a low corrosion current density and a high polarisation resistance indicated a good corrosion resistance of the material [52]. It is observed that MgHAp samples did not show good corrosion resistance evident from the Table 3a.

**Table 3:** a: The corrosion rate of the MgHAp samples as calculated from the Tafel plot. b: The corrosion rate of various AgHAp samples as calculated from the Tafel plot

Sample name	E_corr_ (mV)	I_corr_ (µA/cm^2^)	Corrosion rate (mmpy)
a			
Pure HAp	−476	233.02	60.94
1 wt% MgHAp	−578	699.8	178.10
2 wt% MgHAp	−549	255.8	65.39
3 wt% MgHAp	−521	61.094	15.70
4 wt% MgHAp	−548	56.623	14.60
b			
Pure HAp	−476.0	233.02	60.94
1 wt% Ag doped HAp	−497.1	48.66	14.11
2 wt% Ag doped HAp	−485.8	16.59	4.704
3 wt% Ag doped HAp	−257.8	2.77	0.7672
4 wt% Ag doped HAp	−472.1	36.97	10.034

The Tafel plot for AgHAp samples is presented in Fig. 5b. All the silver doped samples showed a significant shift in the corrosion potential values to the positive side indicating a better corrosion resistance as evident from the corrosion rate values listed in Table 3b [53]. The i_corr_ values decreased with the Ag concentration [52]. The reason of silver having very low corrosion rate is because Ag has very high positive standard electrode potential (+0.799 V) (the second highest followed by Au) making it very difficult to oxidise [54]. When coming in contact with air, it forms a protective layer of silver sulfide making it very difficult to oxidise thus improving its corrosion resistance.Thus, it is observed that AgHAp samples have better corrosion resistance than MgHAp samples. Best corrosion resistance is for 2 wt% AgHAp samples.

### Dielectric Properties

**Fig. 6:**
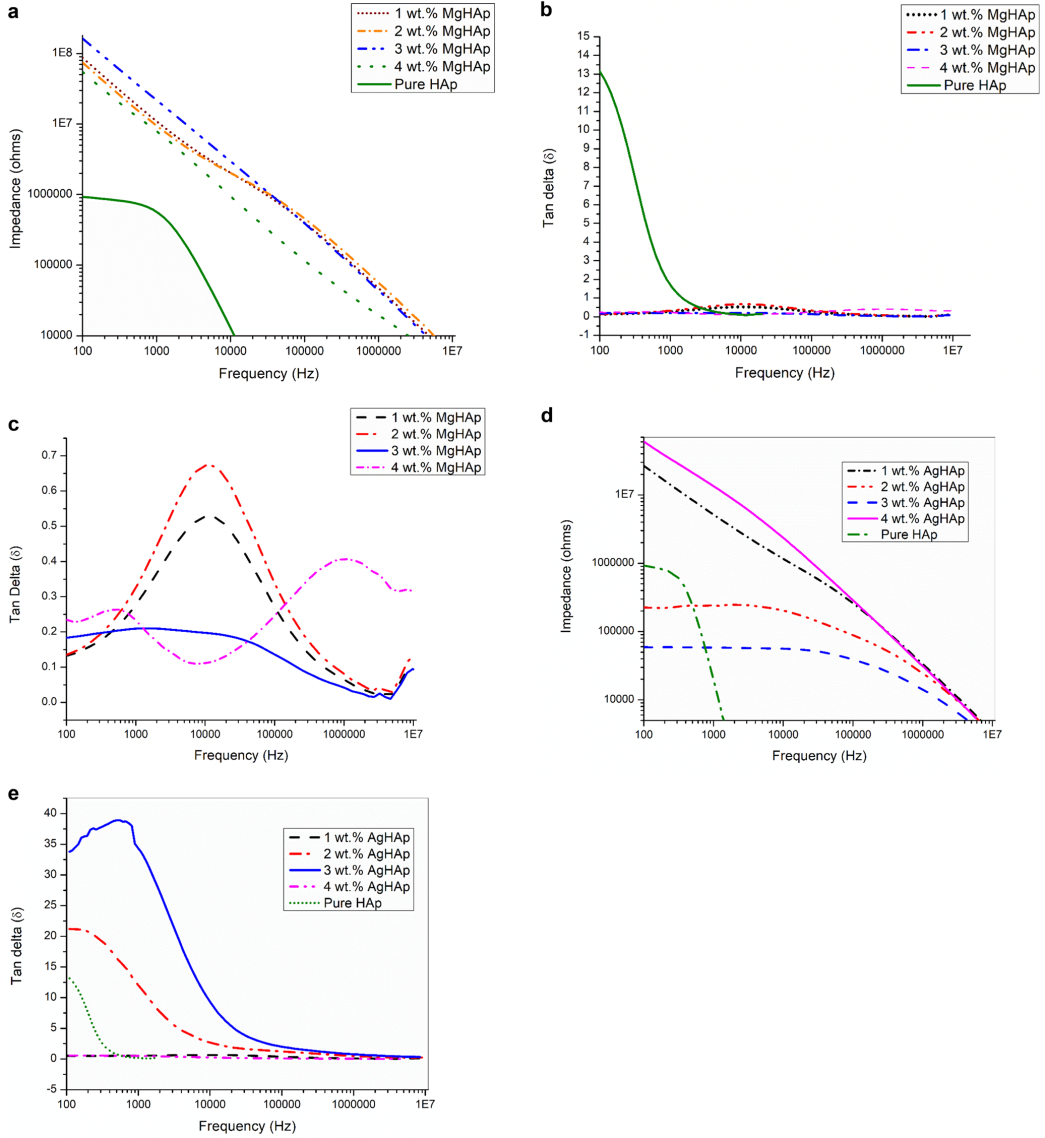
**a** The Bode plot for MgHAp samples. **b** The dielectric loss for MgHAp samples. **c** The magnified image for dielectric loss for MgHAp samples. **d** The Bode plot for all AgHAp samples. **e** The dielectric loss for AgHAp samples

The dielectric material absorbs energy from the alternating electric field according to its capacity; it stores a part of the energy and loses the remaining energy as heat at specified frequency. The dissipated energy is referred to as dielectric loss. The impedance and the dielectric loss for MgHAp samples is represented in Fig. 6b,c. The crystal structure, applied AC frequency, temperature, structure defects, flaws in the crystal lattice, microstructures, dislocations, and other variables all impact the dielectric loss. Therefore, by choosing the appropriate material, the dielectric loss may be reduced. Low frequency causes the polarisation to be dominated by charge carriers, which means that more energy is needed for rotating the dipoles, increasing the dielectric loss. In each instance, the dielectric loss rises sharply at lower frequencies and falls off progressively as frequency rises. The concentration of dopants and their composition determine the variation in dielectric loss. Therefore, the dielectric loss can be minimised by using proper material. The dielectric loss goes on decreasing with the increase of frequency and the increase in the concentration of dopant material Thus 3 wt% MgHAp shows the least dielectric loss with the increasing frequency followed by 2 wt% Mg HAp, 1 wt% MgHAp and 4 wt% MgHAp. At low frequency 10^4 ^Hz, 2 wt% MgHAp shows the highest dielectric loss which can be explained by the oscillations of the dipole which causes the substantial increase in dielectric loss at low frequencies. The polarisation of the ions stops at a higher frequency. Thus 3 wt% MgHAp shows the least dielectric loss comparatively. As a result, the ion dipole in HAp is not rotated using the energy. As a result, the dielectric loss decreases. Doping of Mg ions increases the dielectric constant [55]. At low frequency, the dielectric constant was high due to the ionic polarisation but subsequently reduced by lagging of dipole constant electric field [56]. At lower frequency, it was enhanced in the incorporated samples perhaps due to the raised ionic polarisation [55]. Reduction of permittivity at higher frequency is possibly due to the lagging of dipole oscillations with the applied electric field. As the Mg concentration increases, both octahedral and interstitial sites gets occupied and at low frequency there was strong orientation of dipoles and at higher frequency, the orientation of the dipoles are disturbed [55]. At lower frequency, values of impedance as shown in Fig. 6a are enhanced in the incorporated samples due to the increased ionic polarisation. Reduction in impedance with the increasing frequency was possibly due to the lagging of dipole oscillation with the applied field. As the concentration of Mg^2+^ ions increase by replacing the Ca^2+^ sites, the values of impedance also decrease.

The impedance value for all AgHAp samples is represented in Fig. 6d. The impedance tends to decrease with increasing frequency. Impedance is indirectly proportional to conductivity. Therefore, decrease in impedance value for all AgHAp samples implied better conductivity. Researchers have studied the relationship between tissue electrical characteristics and development, healing and regeneration, focusing in particular on frog and rat studies of lost limb regeneration and partial limb regeneration triggered by electrical impulses [57]. The dielectric loss as shown in Fig. 6e is a function of frequency and it was seen that as dielectric loss changes with Ag^+^ doping and decreased steadily with increasing frequency (2.5 × 10^4 ^Hz for 2 wt% Ag and 3 wt% AgHAp) and mostly attains a constant value for 1 wt% and 4 wt% AgHAp till the frequency of 10^7^ Hz [58]. Due to the high dielectric polarising ability of the Ag, the dielectric loss decreases with increase in Ag content [59]. Dielectric loss for 4 wt% AgHAp and 3wt% MgHAp is minimum as seen from the graphs. However, impedance and corrosion rate for 3wt% AgHAp is minimum. This can be correlated with crystallite size and pore size.

### FESEM analysis

**Fig. 7:**
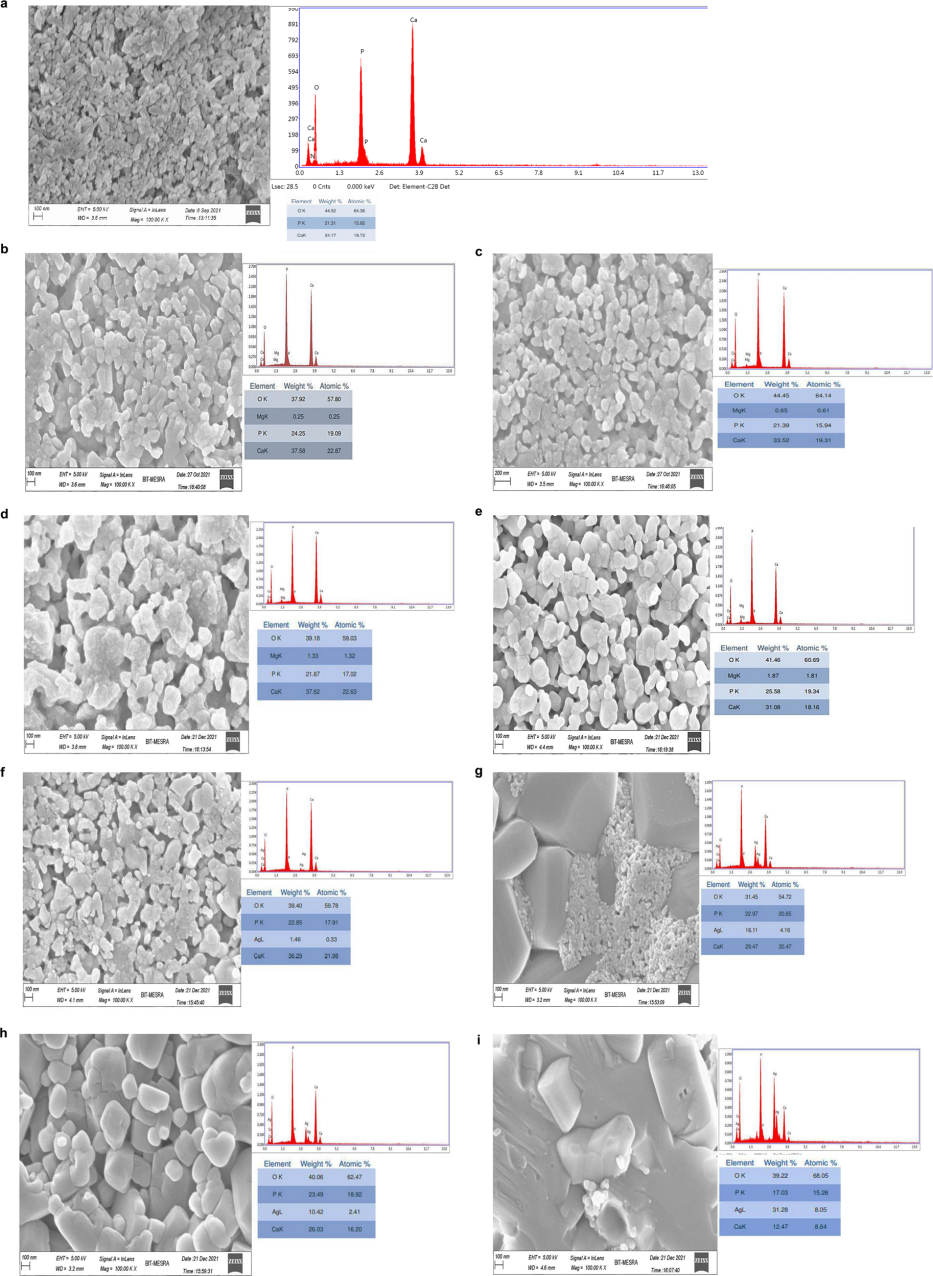
**a** The morphology of pure HAp sample. **b** The morphology of 1 wt% MgHAp. **c** The morphology of 2 wt% MgHAp. **d** The morphology of 3 wt% MgHAp. **e** The morphology of 4 wt% MgHAp. **f** The morphology of 1 wt% AgHAp. **g** The morphology of 2 wt% AgHAp sample. **h** The morphology of 3 wt% AgHAp sample. **i** The morphology of 4 wt% AgHAp sample

The morphology and elemental composition of the samples were studied using FESEM coupled with EDX. The surface morphology of pure HAp and various MgHAp is illustrated in Fig. 7a–e. The morphology of pure hydroxyapatite as shown in FESEM micrograph revealed the particles are granular shaped and are small in size. The MgHAp image shows that the particles are loosely agglomerated.The figure demonstrated that the samples showed agglomeration of small particles [26]. The particles have globular or spherical shape. The agglomeration increases as the weight percent of magnesium is increased.

The FESEM images for various weight percent AgHAp are shown in Fig. 7f–i. As the concentration of Ag in HAp increased, the size of the particles also increases due to the agglomeration. The morphology as seen from the images showed that when the concentration of Ag is increased, the small particles are agglomerated to form aggregates. As concentration of Ag increased, the grain boundary disappears as evident from the FESEM images. In case of 4 wt% AgHAp, no pores were seen with high extent of agglomeration [42].

The elemental composition for HAp, MgHAp and AgHAp are analysed using EDX. The ratio of Ca/P, (Ca+Mg)/P and (Ca+Ag)/P was also measured. From the EDX graph, we see that the main elements present in pure HAp are Ca, P, O, small amount of N, Ag and Mg in case of magnesium and silver doped samples. The ratio for all the doped samples ranged in between 1.59 to 1.67. From the table, the Ca/P ratio in pure HAp was 1.60 which was close to the ratio found in stoichiometric HAp, that is 1.67. However, it was found out that the powders and the pellets having ratio in the range 1.50 to 1.667 have structures similar to that of HAp [60]. The ratio obtained for HAp coated implants lie within the range of 1.67 to 1.76 [61]. The increase in the ratio could probably be the result of carbonate or hydroxyl substitution in place of phosphate groups [62]. Bigger grains are observed in AgHAp samples with smaller number of pores. Comparatively, in MgHAp smaller grains are observed with number of pores.

### BET studies

Pore size and porosity are very important factors in determining the biocompatibility of the implants. The implant must resemble the natural bone not only in the composition but it should have similarity with the structural morphology also. Hence, it is very important to prepare a biomaterial with interconnected pores to have good porosity; this is to make a good synthetic bone substitute [63]. The existence of pores would be accompanied by the reduction in the mechanical stability of calcium-phosphate based ceramics [64]. The size of the pore for pure HAp and the different wt% MgHAp and AgHAp are illustrated in the Table 4a, b, respectively. The data showed that the size of the pores is in the nanometre range with no gradual increase or decrease in the pore size. The maximum pore size was observed in 4 wt% MgHAp. The surface area and the pore volume of the pores is also listed in the same Table 4a, b.In AgHAp, the maximum pore radius was found for 2 wt% AgHAp. This can also be seen FESEM micrographs. From BET table of AgHAp and MgHAp, it can be observed that surface area is more for MgHAp than AgHAp.

**Table 4:** a: The pore size and pore volume for various MgHAp samples. b: The pore size and pore volume for the various AgHAp samples

Sample Name	Pore volume (cc/g)	Average Pore Radius (nm)	Surface area (m^2^/g)
a			
Pure HAp	0.011	1.457	3.293
1 wt% MgHAp	0.015	1.807	4.396
2 wt% MgHAp	0.014	1.814	4.075
3 wt% MgHAp	0.112	1.816	3.305
4 wt% MgHAp	0.018	1.806	4.437
b			
Pure HAp	0.0115	1.457	3.293
1 wt% AgHAp	0.0072	1.807	3.197
2 wt% AgHAp	0.0021	2.291	3.108
3 wt% AgHAp	0.0052	1.861	3.082
4 wt% AgHAp	0.0068	2.274	3.062

### Thrombogenicity studies

Another surface property of material which may be effectively modified through metal doing is thrombogenicity. Thrombogenic materials are clot producing materials. Sometimes, thrombogenic materials with rough surfaces are used to promote clotting in porous interstices to prevent initial leaking of blood and to allow later tissue ingrowth through the pores of vascular implants. For hard tissue replacement, thrombogenic materials are used. More clotting on the surface enhances new bone formation. Whereas for soft tissue replacement, non-thrombogenic materials are required.

**Fig. 8:**
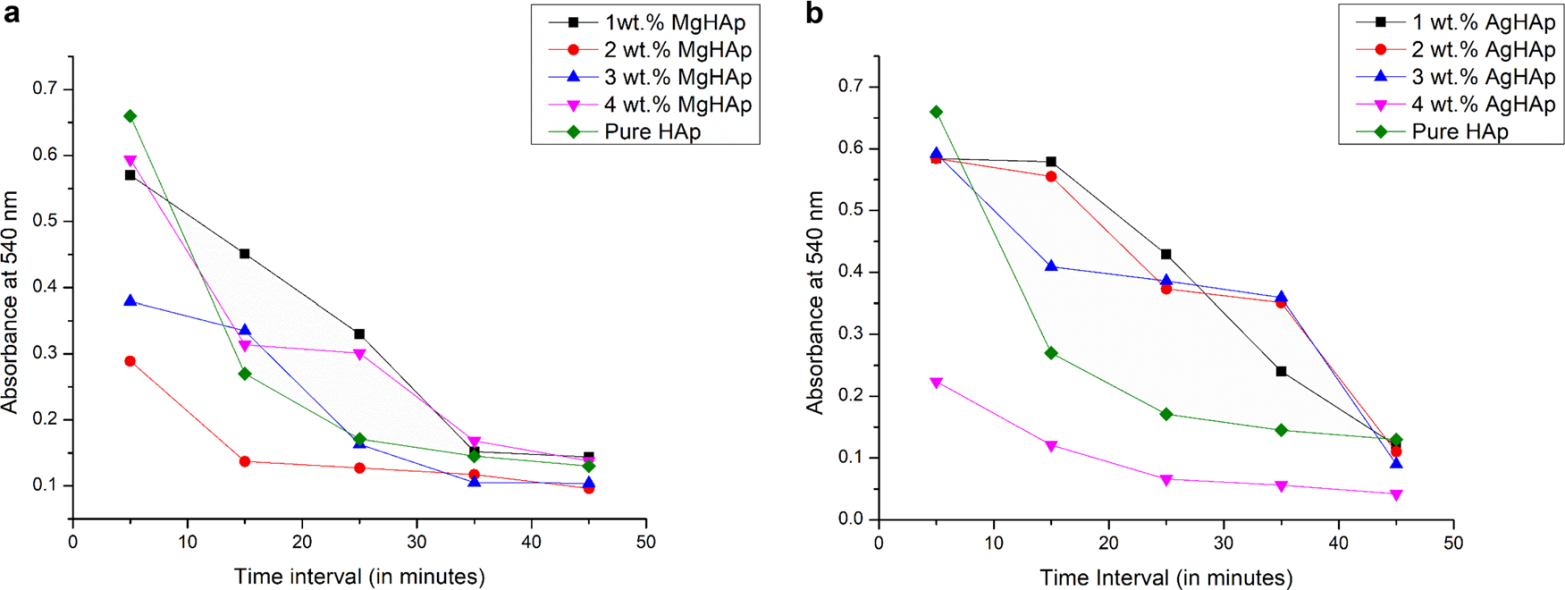
**a** The thrombus behaviour of MgHAp samples. **b** The thrombus behaviour of AgHAp samples

Figure 8a, b shows the thrombus behaviour of the MgHAp and AgHAp respectively. All the samples showed moderate thrombogenic behaviour. The clotting values at different incubation time interval for MgHAp and AgHAp was represented in Table 5a, b, respectively. The concentration of haemoglobin (Hb) was checked at 540 nm because the absorbance value for haemoglobin was at 540 nm [65]. The experiment is about concentration of free haemoglobin after incubation for different time intervals. The size of the clot was found to be inversely related to the absorbance value [16]. There are two factors on which thrombogenicity depends. Firstly, the surface roughness and secondly the wettability. Surface roughness showed a direct relation with the thrombogenicity while wettability shared an inverse relation with the thrombogenicity that is hydrophilic surfaces showedless thrombogenicity. It was found that the thrombus behaviour was the best for 2 wt% MgHAp and 4 wt% AgHAp.

**Table 5:** a: The clotting time of various weight percents MgHAp samples. b: The clotting time of different weight percents of AgHAp samples

Sample Name	5 min	15 min	25 min	35 min	45 min
a					
Pure HAp	0.661	0.27	0.171	0.145	0.139
1 wt% MgHAp	0.571	0.451	0.332	0.152	0.144
2 wt% MgHAp	0.289	0.137	0.127	0.117	0.096
3 wt% MgHAp	0.379	0.335	0.163	0.105	0.104
4 wt% MgHAp	0.594	0.314	0.301	0.168	0.138
b					
Pure HAp	0.661	0.27	0.171	0.145	0.139
1 wt% AgHAp	0.584	0.579	0.429	0.241	0.122
2 wt% AgHAp	0.584	0.555	0.373	0.351	0.112
3 wt% AgHAp	0.592	0.409	0.386	0.359	0.097
4 wt% AgHAp	0.224	0.121	0.662	0.056	0.042

In this present comparative study of MgHAp and AgHAp, following important points were concluded. Crystallite size is inversely related to hardness. Thus, smaller the crystallite size, harder will be the samples. Thus, for all MgHAp samples, the crystallite size was found to decrease as the concentration of Mg was increased. In case of AgHAp samples, as the concentration of Ag was increased, the crystallite size also increased. Thisimplied that the hardness of all the AgHAp samples decreased with increase in dopant concentration. From Vicker’sMicroindetertest, it was also confirmed that MgHAp samples exhibited higher hardness in comparison to AgHAp samples. Hardness for all wt% MgHAp improved and for 4 wt% MgHAp, hardness was found to be the best. This is attributed to the smaller ionic radii of Mg^2+^ than Ag^+^ ions. There was shifting of Raman peak for both AgHAp and MgHAp which confirmed the incorporation of Ag and Mg in the respective samples. Also, it was concluded from Raman peak that as the concentration of the dopant was increased, stability of AgHAp and MgHAp increased whichwas confirmed from thedecrease in the intensity of the vibrational mode of phosphate at 1120- 1122 cm^−1^ with the increase in the dopant concentration. Impedance and dielectric loss for all MgHAp and AgHAp samples was found to decrease in comparison to pure HAp. Initially, the impedance was high for all weight percent MgHAp samples at 100 Hz frequency. But with increase in the frequency, the impedance decreased and the lowest impedance was seen for 4 wt% MgHAp. The dielectric loss for 2 wt% MgHAp is maximum followed by 1 wt% MgHAp. The least dielectric loss was observed for 3 wt% MgHAp which is attributed to the low ionic polarisation at higher frequency.Highest impedance was observed for 4 wt% AgHAp at lower frequency followed by 1 wt%, 2 wt% and 3 wt% AgHAp. The dielectric loss was found to be maximum for 3 wt% followed by 2 wt% AgHAp. The dielectric loss for 1 and 4 wt% AgHApare found to be least and remained constant with increase in frequency.The pore radius was found to increase with increase in dopant concentration in comparison to pure HAp. In case of MgHAp samples, the average pore radius is found to increase due to decrease in crystallite size but for 4 wt% the average pore radius has decreased which may be attributed to the higher concentration of Mg. The average pore radius for 1 wt% and 2 wt% AgHAp has found to increase but in case of 3 wt% AgHAp, the average pore radius has decreased. MgHAp exhibited poor corrosion resistance whereas the AgHAp samples showed very good corrosion resistance and the lowest corrosion rate was exhibited by 3 wt% AgHAp. There was not much change in the thrombogenic behaviour of all the AgHAp and MgHAp samples and all the samples showed moderate clotting behaviour.

## Conclusions

It is impressive to understand the dopant ionic size effects on the properties of HAp. As in the HAp, main constituent is calcium ion, a smaller size dopant like Mg ion and on bigger size dopant like Ag ion are chosen and changes in HAp microstructural, mechanical, physiochemical & biocompatible properties are studied and described in this paper. Different concentration of Mg doped HAp and Ag doped HAp are synthesised successfully using sol-gel method. It is evident from the XRD analysis that the crystallinity of MgHAp has decreased significantly with the increase in the concentration of Mg ion in the HAp matrix. This inferred that the amorphous behaviour starts to predominate. In contrast to MgHAp samples, the crystallite size for various AgHAp samples shows drastic increase confirming that incorporation of Ag^+^ ions in the HAp matrix increases the crystallinity of the samples. There is an inverse relation between hardness and crystallite size. As the size decreases, the hardness will increase so is seen in case of 4 wt% MgHAp with crystallite size of 17.89 nm. From the values of crystallite size, it can be inferred that MgHAp samples have high mechanical strength than AgHAp samples. This was confirmed from hardness test. The maximum hardness is shown for 4 wt% MgHAp with a value of 56.18 GPa while 1 wt% MgHAp shows the least hardness with a value of 39.17 GPa.4 wt% AgHAp shows the lowest hardness with a value of 36.79 GPa wherein 1 wt% AgHAp shows the highest hardness with a value of 45.94 GPa. The morphology analysis confirmed that with the increase in the weight percent of Mg, the size of the grains decreased. Reverse trend was observed in case of AgHAp samples. All the samples had Ca/P ratio in the range of 1.5 to 1.67confirming that HAp phase has been formed. The corrosion resistance analysis showed that AgHAp had the highest corrosion resistance and the best is shown by 3 wt% AgHAp with corrosion rate value of 0.7671 mmpy while MgHAp is highly prone to corrosion with a corrosion rate value 14.60 mmpy for 4 wt% MgHAp. Dielectric loss for 4 wt% AgHAp and 3wt% MgHAp is minimum which is in agreement with the corrosion results. Both MgHAp and AgHAp showed moderate thrombus behaviour. The best result for thrombogenicity was shown by 2 wt% MgHAp samples. Pore size holds an inverse relation with the mechanical properties. Highly porous surfaces exhibit lower hardness.The pore radius was found to be maximum for AgHAp samples consequently the hardness for AgHAp samples was also found to be lesser than the MgHAp samples. MgHAp has overall best properties but needs to be alloyed with some other metals to improve the corrosion resistance while the in-vitro thrombogenicity values showed good results for both MgHAp and AgHAp samples.
